# From Gym to App: integrating digital monitoring to enhance student engagement in physical education

**DOI:** 10.1080/21642850.2026.2741524

**Published:** 2026-10-14

**Authors:** Stefan Alecu, Gheorghe Adrian Onea

**Affiliations:** a Faculty of Physical Education and Mountain Sports, Transilvania University, Brasov, Romania

**Keywords:** Physical activity, digital technology, student engagement, motivation, physical education

## Abstract

This study examines the effect of integrating a digital fitness application into university physical education on students’ physical activity, motivation, and self-perceptions. A quasi-experimental design was conducted over 14 weeks with 1,614 undergraduate students, divided into an experimental group (*n* = 812) and a control group (*n* = 802). Both groups attended weekly physical education sessions, while the experimental group also used the Google Fit application to monitor activity and achieve at least 150 Heart Points per week. Physical activity and motivation were assessed using an IPAQ-derived activity composite and a BREQ-3 aggregate, alongside measures of perceived physical competence and body image. Both groups showed improvements, but the experimental group reported significantly greater changes. Physical activity increased by 0.66 points in the experimental group (Cohen's dz = 1.42) and by 0.18 points in the control group (dz = 0.40), while motivation increased by 0.56 points (dz = 1.63) and 0.12 points (dz = 0.40), respectively. Both outcomes were derived from 5-point response scales. Cluster-adjusted differences in change were statistically significant across all variables (*p* < 0.001). The findings are consistent with a beneficial effect of the multicomponent digitally supported intervention, while the quasi-experimental design limits causal attribution.

## Introduction

Physical inactivity among young adults remains a major public health concern, with university students identified as a particularly vulnerable group (Buckworth & Nigg, 2004; Deliens et al., 2015; Haase et al., 2004; Irwin, 2004). The transition to higher education often leads to a decline in physical activity due to changes in routine, increased academic pressure, and reduced structured opportunities for movement (Bray & Born, 2004; Caspersen et al., 2000; Nelson et al., 2008). As a result, many students fail to meet recommended activity levels, which has implications for both physical and mental health (Guthold et al., 2018; Stefanica et al., 2025; WHO, 2020). University physical education programmes aim to address this issue by promoting regular participation in physical activity. However, their impact remains limited when engagement is restricted to scheduled sessions (McKenzie & Lounsbery, 2013; Hills et al., 2015; Kriemler et al., 2011). Students often participate during class time but do not translate this engagement into independent behaviour outside the institutional context (Keating et al., 2005; Leslie et al., 1999). This highlights the need for strategies that support continuous engagement and encourage self-directed activity.

Digital technologies offer new opportunities to reshape how students interact with physical activity. Mobile health applications and wearable devices provide real-time feedback, track progress, and allow users to set personalised goals (Piwek et al., 2016; Ridgers et al., 2016). These features can enhance self-awareness and support behaviour change by making activity patterns more visible and measurable (Lyons et al., 2014; Patel et al., 2015). Evidence suggests that interventions that combine monitoring with goal-setting are more effective in increasing physical activity (Kryeziu et al., 2023; Michie et al., 2017). The use of digital tools also aligns with broader shifts in educational practice, where learning extends beyond formal settings and becomes more individualised (Selwyn, 2016). In physical education, this shift supports the development of lifelong activity habits rather than short-term participation (Ennis, 2017; Kirk, 2010). Technology can facilitate this process by enabling students to engage with physical activity in their own time and context (Badau et al., 2025).

Motivation plays a central role in sustaining physical activity behaviour. Research shows that students are more likely to remain active when they experience a sense of autonomy and competence (Hagger & Chatzisarantis, 2007; Teixeira et al., 2012). Digital tools can support these processes by providing feedback and allowing users to track their own progress. This can lead to increased engagement and more consistent participation over time (Kerner & Goodyear, 2017; Lewis et al., 2016). In addition to behavioural outcomes, physical activity is closely linked to psychological factors such as self-perception and body image (Fox, 2001; Hausenblas & Fallon, 2006). Students who engage regularly in physical activity tend to report higher levels of perceived competence and more positive body image (Babic et al., 2014; Crocker et al., 2003). Interventions that increase activity levels may therefore also influence how students perceive themselves. Digital interventions in physical activity and physical education are increasingly studied; however, fewer studies have embedded continuous app-based monitoring within routine university physical education as part of a teacher-supported, multicomponent intervention while reporting both behavioural and self-perception outcomes (Dennison et al., 2013; Direito et al., 2015; Rand et al., 2026). From a Self-Determination Theory perspective, self-monitoring may support autonomy, progress feedback and attainable targets may support competence, and teacher support may contribute to relatedness; these mechanisms are theoretically plausible but were not directly measured in the present study (Ahmadi et al., 2023).

The present study addresses this gap by investigating changes associated with integrating a multicomponent digitally supported intervention into university physical education. The intervention combines weekly face-to-face sessions with continuous activity monitoring, allowing students to engage with physical activity beyond the classroom. This approach aims to increase both the quantity and quality of physical activity, while also supporting motivation and self-perception. By examining changes in physical activity, motivation, and psychological outcomes, the study provides a comprehensive evaluation of the intervention. It also contributes to current debates on the role of technology in education and its potential to support more effective and sustainable approaches to physical activity promotion. We hypothesised that students in the experimental condition would show greater pre-to-post improvements in physical activity, motivation, perceived physical competence, and body image than students in the control condition.

## Materials and methods

This study employed a quasi-experimental design to investigate the effects of integrating a digital fitness application into university physical education on students’ participation, motivation, and engagement in physical activity. The research was conducted over a 14-week monitoring period from 24 February to 8 June 2025, including examination and holiday periods, and combined traditional gym-based instruction with digitally supported physical activity. Heart Points were recorded throughout the 14-week monitoring window, while face-to-face feedback was delivered during scheduled physical education sessions.

The sample consisted of 1,614 undergraduate students (male and female), aged between 19 and 25.9 years (M = 21.72, SD = 1.49), enroled in the first and second years of study across multiple faculties at Transilvania University of Brașov, Romania. Students from programmes related to Physical Education and Sport, Sport and Motor Performance, and Physiotherapy and Special Motricity were excluded in order to minimise bias associated with higher initial test levels of physical activity and prior professional training. Participation was voluntary and based on informed consent, and all procedures were conducted in accordance with institutional ethical standards and the Declaration of Helsinki.

Participants were allocated at the class level into an experimental group (*n* = 812; 27 classes) and a control group (*n* = 802; 27 classes), preserving the natural structure of teaching activities. Class size was approximately 30 students (28–31 in the experimental condition and 29–30 in the control condition). No formal randomisation procedure was documented; the design was therefore treated as non-randomised and quasi-experimental. Both groups participated in one 90-minute physical education session per week throughout the semester. These sessions included a combination of warm-up and mobility exercises, as well as general fitness training targeting strength, endurance, and coordination.

In addition to the standard programme, students in the experimental group used the Google Fit mobile application to monitor their daily physical activity. The application was selected due to its accessibility and its ability to track activity levels based on Heart Points, an intensity-based metric aligned with World Health Organisation recommendations. Students were instructed to use the application daily and to achieve a minimum of 150 Heart Points per week. Teaching staff monitored activity levels and provided regular feedback during physical education sessions. Short discussions were also included to support goal setting and increase awareness of physical activity behaviour. Accordingly, the intervention was multicomponent, combining app-based self-monitoring, an explicit weekly activity target, instructor monitoring, regular feedback, and brief goal-setting discussions; the independent contribution of Google Fit cannot be isolated from these components.

Data were collected at initial test (Week 1) and immediately following the 14-week intervention (Week 14). Self-reported physical activity levels were assessed using seven IPAQ-derived activity items, while motivation for physical activity was measured using the 24-item Behavioural Regulation in Exercise Questionnaire (BREQ-3). For the present dataset, the seven activity items were recorded on 5-point response scales and averaged to form an activity composite (range 1-5); this is not the conventional IPAQ-SF MET-min/week score and is therefore reported as an IPAQ-derived activity composite. The 24 BREQ-3 items were recorded on 5-point Likert scales and averaged to form the study's aggregate motivation score. Because BREQ-3 is multidimensional, this aggregate is interpreted descriptively rather than as a validated unidimensional BREQ-3 total score. Perceived physical competence and body image were each assessed with a single 5-point self-report item. In the present sample, Cronbach's alpha was 0.79 at baseline and 0.87 at Week 14 for the seven-item activity composite, and 0.92 and 0.95, respectively, for the 24-item BREQ-3 aggregate. Internal-consistency coefficients are not applicable to the single-item competence and body-image measures.

For the experimental group, objective physical activity data were obtained using Google Fit, Version 2025.03.12.00, (Google LLC, Mountain View, CA, USA), a mobile health application that enables continuous monitoring of activity patterns. Particular attention was given to Heart Points, an intensity-based metric aligned with World Health Organisation guidelines, whereby one point is awarded for each minute of moderate-intensity activity and two points for each minute of vigorous-intensity activity. This metric provides a more comprehensive representation of physical activity by incorporating both duration and intensity. In the experimental group, all 812 participants had Heart Points data for all 14 weeks (11,368 participant-weeks). The weekly target of at least 150 Heart Points was achieved in 89.1% of participant-weeks; 75.9% of participants met the target in at least 80% of weeks, and 47.2% met it in all 14 weeks.

Statistical analyses were performed using IBM SPSS Statistics for Windows, Version 26.0 (IBM Corp., Armonk, NY, USA), with supplementary cluster-adjusted analyses based on the participant-level dataset. Descriptive statistics (means and standard deviations) were calculated for all variables. Baseline between-group comparisons and within-group changes are retained as secondary descriptive analyses. The primary intervention contrast was the between-group difference in pre-to-post change. Because allocation occurred by class, change-score regression models used cluster-robust standard errors at the class level (54 classes); with two measurement occasions, the condition coefficient estimates the same difference-in-change contrast as a time-by-group interaction. For descriptive interpretation of within-group pre-to-post changes, Cohen's dz was calculated as the mean change divided by the standard deviation of the individual change scores. Cohen's d was calculated from individual change scores, with 95% confidence intervals obtained by resampling classes. Sensitivity models additionally adjusted for age, sex, year of study, and faculty. Statistical significance was set at *p* < 0.05. No formal a priori power calculation determined recruitment; the sample was defined pragmatically by the 54 eligible classes available during the study period. A retrospective design-sensitivity calculation using a conservative ICC of 0.05 and approximately 30 students per class gives a design effect of about 2.45 and an effective sample size of approximately 659, providing 80% power to detect an effect of about d = 0.22 at alpha = 0.05 (Rutterford et al., 2015).

The study received approval from the Ethics Committee of the Faculty, Transilvania University of Brasov (approval no. 819/12.02.2025), and all data were anonymised to ensure confidentiality and compliance with data protection regulations.

## Results

Data from both the experimental and control groups were analysed at baseline and after the 14-week intervention. The final analytic cohort comprised all 1,614 participants from 54 classes (812 experimental; 802 control), with complete baseline and Week 14 questionnaire data. The groups were descriptively similar in age (21.64 + /- 1.48 vs 21.80 + /- 1.49 years), female proportion (53.6% vs 55.1%), and first-year proportion (55.8% vs 56.9%). All 812 participants in the experimental condition had complete Heart Points records across the 14-week monitoring period.

**Table 1.** Descriptive statistics of study variables at the initial test.

Variable	Group	X	SD	CV%	95% CI		Kurtosis	*p*
					LL	UL		
**Activity composite (1-5)**	Experimental	2.80	0.56	20.17	2.76	2.84	−0.01	0.595
	Control	2.78	0.59	21.32	2.74	2.82	−0.39	
**BREQ-3 aggregate**	Experimental	2.96	0.56	18.95	2.92	3.00	−0.19	0.976
	Control	2.96	0.54	18.11	2.93	3.00	−0.24	
**Physical competence**	Experimental	3.00	0.81	27.19	2.94	3.05	0.03	0.318
	Control	3.04	0.84	27.64	2.98	3.09	−0.03	
**Body image**	Experimental	2.91	0.92	31.49	2.84	2.97	−0.22	0.274
	Control	2.86	0.91	31.85	2.79	2.92	−0.28	

Note: X = mean; SD = standard deviation; CV% = coefficient of variation; CI = confidence interval; LL = lower limit; UL = upper limit; IPAQ-derived activity composite = mean of seven 5-point activity items; BREQ-3 aggregate = mean of 24 BREQ-3 items; *p* = baseline between-group test.

Table 1 shows that the experimental and control groups were descriptively similar at the initial test, with no statistically significant baseline differences across the four study outcomes (*p* > 0.05). Mean differences were small and the 95% confidence intervals overlapped between groups. Kurtosis values ranged from −0.39 to 0.03, indicating no marked distributional departures.

These baseline tests indicate no detected differences in the measured outcomes but do not establish formal statistical equivalence or eliminate potential confounding in a quasi-experimental design.

**Table 2.** Initial test comparison between experimental and control groups.

Variable	X (Exp)	SD (Exp)	X (Ctrl)	SD (Ctrl)	*t*	*p*
**Activity composite (1-5)**	2.80	0.56	2.78	0.59	0.53	0.595
**BREQ-3 aggregate**	2.96	0.56	2.96	0.54	0.03	0.976
**Physical competence**	3.00	0.81	3.04	0.84	−1.00	0.318
**Body image**	2.91	0.92	2.86	0.91	1.10	0.274

Note: X = mean; Exp = experimental group; Ctrl = control group; SD = standard deviation; activity composite = mean of seven 5-point activity items; BREQ-3 aggregate = mean of 24 BREQ-3 items; t = independent-samples t-test; *p* = two-sided significance value.

Table 2 shows no statistically significant baseline group differences in the four study outcomes (all *p* > 0.27). These non-significant tests are not interpreted as evidence of formal equivalence.

**Table 3.** Within-group changes from initial test to final test.

Variable	Group	X (IT)	X (FT)	Delta X	dz	*t*	*p*
**Activity composite (1-5)**	Experimental	2.80	3.46	+0.66	1.42	40.60	<0.001
	Control	2.78	2.96	+0.18	0.40	11.36	<0.001
**BREQ-3 aggregate**	Experimental	2.96	3.52	+0.56	1.63	46.39	<0.001
	Control	2.96	3.08	+0.12	0.40	11.43	<0.001
**Physical competence**	Experimental	3.00	3.40	+0.41	0.48	13.67	<0.001
	Control	3.04	3.09	+0.05	0.06	1.67	0.096
**Body image**	Experimental	2.91	3.27	+0.36	0.39	11.21	<0.001
	Control	2.86	2.96	+0.10	0.11	3.08	0.002

Note: IT = initial test (Week 1); FT = final test (Week 14); Delta X = mean pre-to-post change; activity composite = mean of seven 5-point activity items; BREQ-3 aggregate = mean of 24 BREQ-3 items; dz = Cohen's standardised within-group mean change, calculated using the standard deviation of individual change scores; t = paired-samples t-test; *p* = two-sided significance value.

Table 3 presents secondary within-group changes. In the experimental group, mean changes were + 0.66 for the activity composite, + 0.56 for the BREQ-3 aggregate, + 0.41 for perceived physical competence, and + 0.36 for body image (all *p* < 0.001). In the control group, changes were + 0.18, + 0.12, + 0.05, and + 0.10, respectively; the competence change was not statistically significant (*p* = 0.096).

The experimental group showed larger mean changes across all four outcomes; the formal between-condition difference in change, accounting for class-level clustering, is reported in Table 4.

Objective monitoring in the experimental condition showed a mean of 191.1 Heart Points per week; mean Heart Points increased from 147.7 in Week 1 to 227.8 in Week 14. The weekly target was met in 89.1% of participant-weeks.

**Table 4.** Primary cluster-adjusted between-group differences in pre-to-post change.

Variable	Delta Exp	Delta Ctrl	DeltaDelta	95% CI	Cohen d	95% CI d	*p*
**Activity composite**	0.664	0.181	0.483	0.446–0.520	1.05	0.97–1.14	<0.001
**BREQ-3 aggregate**	0.557	0.122	0.436	0.401–0.470	1.35	1.23–1.48	<0.001
**Physical competence**	0.406	0.051	0.355	0.263–0.447	0.41	0.31–0.51	<0.001
**Body image**	0.365	0.102	0.262	0.174–0.351	0.28	0.18–0.37	<0.001

Note: Delta Exp/Ctrl = mean pre-to-post change; DeltaDelta = experimental minus control change; CI = 95% confidence interval; d = Cohen's d based on individual change scores. Regression standard errors were clustered by class (54 classes); d confidence intervals were obtained by class-level bootstrap. All *p* values are two-sided.

Table 4 shows statistically significant between-condition differences in pre-to-post change after accounting for class-level clustering (all *p* < 0.001).

The difference in change was 0.483 points for the activity composite (95% CI 0.446-0.520; d = 1.05), 0.436 for the BREQ-3 aggregate (95% CI 0.401-0.470; d = 1.35), 0.355 for perceived physical competence (95% CI 0.263-0.447; d = 0.41), and 0.262 for body image (95% CI 0.174-0.351; d = 0.28). Thus, statistical significance and effect magnitude were not inferred from *p* values alone.

Sensitivity models additionally adjusted for age, sex, year of study, and faculty yielded nearly unchanged intervention estimates (0.482, 0.436, 0.359, and 0.261 points, respectively), supporting the robustness of the primary change-score comparisons to these measured covariates.

## Discussion

The present study examined changes associated with integrating a digital fitness application into university physical education on students’ physical activity, motivation, and self-perceptions. The findings show consistent improvements in the experimental group across all variables, with significantly higher post-intervention scores compared to the control group. These findings are consistent with the growing body of research that highlights the role of digital technologies in enhancing student engagement and promoting active lifestyles (Casey et al., 2016; Goodyear et al., 2019). The larger increase in the activity composite observed in the experimental group aligns with previous studies showing that self-monitoring tools and activity tracking applications can lead to meaningful behavioural changes (Brickwood et al., 2019; Direito et al., 2014). The use of Heart Points as a clear and measurable target appears to have supported students in translating abstract recommendations into concrete actions. Similar findings were reported by Feter et al. (2019), who showed that mobile health interventions improve adherence to physical activity guidelines.

Motivation also improved significantly in the experimental group, which is consistent with Self-Determination Theory (Deci & Ryan, 2000; Ryan & Deci, 2017). The intervention components could plausibly support autonomy through self-monitoring, competence through feedback and goal achievement, and relatedness through teacher-supported discussions. These mechanisms are theoretically relevant to motivation (Ahmadi et al., 2023; Ntoumanis et al., 2021; Teixeira et al., 2012), but basic psychological need satisfaction was not directly measured and the mechanism should therefore be considered hypothetical. The improvements in perceived physical competence and body image further reinforce the psychological impact of the intervention. Students who engage more frequently in physical activity and receive continuous feedback tend to develop more positive self-perceptions (Babic et al., 2014; Lubans et al., 2016). The relatively large changes observed in these variables suggest that digital tools can influence not only behaviour but also how students perceive their own bodies and abilities.

The control group also showed small but significant improvements, which may reflect the effect of regular participation in physical education classes. However, these changes were limited in magnitude, which is consistent with the possibility that traditional formats alone may be less likely to produce substantial behavioural or motivational shifts (Fairclough & Stratton, 2005; Sallis et al., 2012). The addition of a digital component appears to extend learning beyond the classroom and encourages more consistent engagement. These findings are consistent with recent research emphasising the importance of hybrid approaches that combine face-to-face instruction with digital support (Casey & MacPhail, 2018; Kerner & Goodyear, 2017). Such approaches create opportunities for continuous interaction with physical activity, rather than limiting it to scheduled sessions. This is relevant in higher education, where students face time constraints and competing priorities (Keating et al., 2005).

From a pedagogical perspective, the results suggest that digital tools can support a more student-centred approach to physical education. Students become active agents in managing their own activity levels, which aligns with contemporary models of learning that emphasise autonomy and self-regulation (Ennis, 2017; Kirk, 2010). The integration of technology also allows instructors to provide more personalised feedback, which can enhance learning outcomes (Fjellner et al., 2024). At the same time, the findings raise important considerations regarding the implementation of digital interventions. The effectiveness of such tools depends on consistent use and meaningful integration into the curriculum (Imbong, 2019; Selwyn, 2016). Simply introducing an application without clear goals and feedback mechanisms may not produce the same results.

The study also contributes to ongoing debates about the role of technology in education. While some authors express concerns about over-reliance on digital tools (Selwyn, 2016), the present results suggest that, when used strategically, technology can enhance rather than replace traditional teaching practices. The key lies in how these tools are integrated and aligned with pedagogical objectives.

Despite these contributions, several limitations should be acknowledged. The study relies partly on self-reported data, which may introduce bias (Prince et al., 2008), and the activity outcome used an IPAQ-derived 5-point composite rather than conventional MET-min/week scoring. Objective Heart Points were available only for the experimental group, preventing an objective between-group comparison. The class-level allocation was non-randomised, and although analyses accounted for clustering and measured demographic covariates, residual confounding, instructor effects, class schedule, contamination between students, novelty effects, and social desirability cannot be excluded. Perceived competence and body image were assessed with single items, and the BREQ-3 outcome was an aggregate of a multidimensional instrument; these measurement choices limit construct-specific interpretation. The intervention combined app monitoring, an explicit target, instructor monitoring, feedback, and goal setting, so the independent effect of Google Fit cannot be isolated. Although the final analytic cohort had complete outcome data, pre-enrolment screening and exclusion counts were not retained in the analytic dataset. The study was conducted at a single university and had no post-intervention follow-up, so generalisability and persistence of the observed changes remain uncertain.

For higher education, the findings suggest that combining traditional teaching with structured digital support may extend engagement beyond scheduled classes without increasing formal contact hours; implementation should nevertheless consider context, instructor practice, and student access to compatible devices.

Future research should also explore long-term effects. It remains unclear whether the observed improvements persist after the intervention ends. Longitudinal studies could provide insight into the sustainability of behaviour change (Rhodes et al., 2017). Moreover, qualitative approaches could help understand how students experience the use of digital tools and what factors influence their engagement.

The findings are consistent with the integration of digitally supported self-monitoring into physical education as a promising strategy associated with greater physical activity, enhancing motivation, and improving self-perceptions among university students. This study extends previous research by integrating digital monitoring within structured physical education classes, rather than using standalone interventions. This approach extends monitoring beyond scheduled sessions, although longer-term behaviour change was not assessed.

## Conclusions

In this quasi-experimental study, participation in a multicomponent digitally supported physical education programme was associated with greater improvements in students’ physical activity, motivation, and self-perceptions. Students in the experimental condition showed larger pre-to-post changes in activity and motivation than those in the control condition. Cluster-adjusted analyses were consistent with a beneficial intervention effect, while the non-randomised design limits causal attribution. The results highlight the value of extending physical education beyond the scheduled class. The experimental condition combined a 150-Heart-Points target with daily self-monitoring, instructor monitoring, feedback, and goal-setting. Students move from passive participation to active self-management of their physical activity. The present study did not assess whether these changes persisted after the 14-week monitoring period.

The study also found concurrent changes in behavioural and self-perception outcomes. Increases in perceived competence and body image suggest that students not only become more active, but also develop a stronger sense of control and confidence. Long-term adherence was not measured and should be evaluated in follow-up studies.

Overall, the findings support further evaluation of multicomponent digitally supported physical education approaches that extend activity monitoring beyond scheduled classes while preserving teacher support and student self-management.

## Supplementary Material

From gym to app.xlsx From gym to app.xlsx

Ethical Approval FromGymToApp.pdf Ethical Approval FromGymToApp.pdf

## Data Availability

The data that support the findings of this study are available from the corresponding author upon reasonable request.

## References

[cit0001] Ahmadi, A. , Noetel, M. , Parker, P. , Ryan, R. M. , Ntoumanis, N. , Reeve, J. , Beauchamp, M. , Dicke, T. , Yeung, A. , Ahmadi, M. , Bartholomew, K. , Chiu, T. K. F. , Curran, T. , Erturan, G. , Flunger, B. , Frederick, C. , Froiland, J. M. , González-Cutre, D. , Haerens, L. , … Lonsdale, C. (2023). A classification system for teachers' motivational behaviors recommended in self-determination theory interventions. *Journal of Educational Psychology* , *115* (8), 1158–1176. 10.1037/edu0000783

[cit0002] Babic, M. J. , Morgan, P. J. , Plotnikoff, R. C. , Lonsdale, C. , White, R. L. , & Lubans, D. R. (2014). Physical activity and physical self-concept in youth: Systematic review and meta-analysis. *Sports Medicine* , *44* (11), 1589–1601. 10.1007/s40279-014-0229-z 25053012

[cit0003] Badau, D. , Steff, N. , Badau, A. , & Stoica, M. (2025). Enhancing lower-limb simple and choice reaction time and spatial orientation in junior basketball players by implementing fitlight technology in sports training. *Perceptual and motor skills* , *132* (6), 1392–1417. 10.1177/00315125251347793 40446814

[cit0004] Bray, S. R. , & Born, H. A. (2004). Transition to university and vigorous physical activity: Implications for health and psychological well-being. *Journal of American College Health* , *52* (4), 181–188. 10.3200/JACH.52.4.181-188 15018429

[cit0005] Brickwood, K. J. , Watson, G. , OBrien, J. , & Williams, A. D. (2019). Consumer-based wearable activity trackers increase physical activity participation: Systematic review and meta-analysis. *JMIR mHealth and uHealth* , *7* (4), e11819. 10.2196/11819 PMC6484266 30977740

[cit0006] Buckworth, J. , & Nigg, C. (2004). Physical activity, exercise, and sedentary behavior in college students. *Journal of American College Health* , *53* (1), 28–34. 10.3200/JACH.53.1.28-34 15266727

[cit0007] Casey, A. , Goodyear, V. A. , & Armour, K. M. (Eds.). (2016). *Digital Technologies and Learning in Physical Education: Pedagogical cases* (1st ed.). Routledge. 10.4324/9781315670164

[cit0008] Casey, A. , & MacPhail, A. (2018). Adopting a models-based approach to teaching physical education. *Physical Education and Sport Pedagogy* , *23* (3), 294–310. 10.1080/17408989.2018.1429588

[cit0009] Caspersen, C. J. , Pereira, M. A. , & Curran, K. M. (2000). Changes in physical activity patterns in the United States, by sex and cross-sectional age. *Medicine & Science in Sports & Exercise* , *32* (9), 1601–1609. 10.1097/00005768-200009000-00013 10994912

[cit0010] Crocker, P. , Sabiston, C. , Forrestor, S. , Kowalski, N. , Kowalski, K. , & McDonough, M. (2003). Predicting change in physical activity, dietary restraint, and physique anxiety in adolescent girls: Examining covariance in physical self-perceptions. *Canadian journal of public health = Revue canadienne de sante publique* , *94* (5), 332–337. 10.1007/BF03403555 PMC6979849 14577738

[cit0011] Deci, E. L. , & Ryan, R. M. (2000). The “What” and “Why” of goal pursuits: Human needs and the self-determination of behavior. *Psychological Inquiry* , *11* (4), 227–268. 10.1207/S15327965PLI1104_01

[cit0012] Deliens, T. , Deforche, B. , De Bourdeaudhuij, I. , & Clarys, P. (2015). Determinants of physical activity and sedentary behaviour in university students: A qualitative study using focus group discussions. *BMC Public Health* , *15* , 201. 10.1186/s12889-015-1553-4 PMC4349731 25881120

[cit0013] Dennison, L. , Morrison, L. , Conway, G. , & Yardley, L. (2013). Opportunities and challenges for smartphone applications in supporting health behavior change: Qualitative study. *Journal of Medical Internet Research* , *15* (11), e258. 10.2196/jmir.2583 PMC3636318 23598614

[cit0014] Direito, A. , Jiang, Y. , Whittaker, R. , & Maddison, R. (2015). Smartphone apps to improve fitness and increase physical activity among young people: Protocol of the apps for IMproving FITness (AIMFIT) randomized controlled trial. *BMC Public Health* , *15* , 635. 10.1186/s12889-015-1968-y PMC4702326 26159834

[cit0015] Direito, A. , Dale, L. P. , Shields, E. , Dobson, R. , Whittaker, R. , & Maddison, R. (2014). Do physical activity and dietary smartphone applications incorporate evidence-based behaviour change techniques? *BMC Public Health* , *14* , 646. 10.1186/1471-2458-14-646 PMC4080693 24965805

[cit0016] Ennis, C. D. (2017). Educating students for a lifetime of physical activity: Enhancing mindfulness, motivation, and meaning. *Research Quarterly for Exercise and Sport* , *88* (3), 241–250. 10.1080/02701367.2017.1342495 28742426

[cit0017] Fairclough, S. , & Stratton, G. (2005). Physical education's contribution to young people's physical activity levels. *Health Education Research* , *20* (1), 14–23. 10.1093/her/cyg101 15253994

[cit0018] Feter, N. , Dos Santos, T. S. , Caputo, E. L. , & da Silva, M. C. (2019). What is the role of smartphones on physical activity promotion? A systematic review and meta-analysis. *International journal of public health* , *64* (5), 679–690. 10.1007/s00038-019-01210-7 30758514

[cit0019] Fjellner, R. L. , Varea, V. , & Barker, D. (2024). How physical education teachers are positioned in models scholarship: A scoping review. *Physical Education and Sport Pedagogy* , *29* (4), 329–345. 10.1080/17408989.2022.2083098

[cit0020] Fox, K. R. (2001). *The effects of exercise on self-perceptions and self-esteem* (1st ed.). Routledge.

[cit0021] Goodyear, V. A. , Kerner, C. , & Quennerstedt, M. (2019). Young people’s uses of wearable healthy lifestyle technologies; surveillance, self-surveillance and resistance. *Sport, Education and Society* , *24* (3), 212–225. 10.1080/13573322.2017.1375907

[cit0022] Guthold, R. , Stevens, G. A. , Riley, L. M. , & Bull, F. C. (2018). Worldwide trends in insufficient physical activity from 2001 to 2016: A pooled analysis of 358 population-based surveys with 1·9 million participants. *The Lancet Global Health* , *6* (10), e1077–e1086. 10.1016/S2214-109X(18)30357-7 30193830

[cit0023] Haase, A. , Steptoe, A. , Sallis, J. F. , & Wardle, J. (2004). Leisure-time physical activity in university students from 23 countries: Associations with health beliefs, risk awareness, and national economic development. *Preventive medicine* , *39* (1), 182–190. 10.1016/j.ypmed.2004.01.028 15208001

[cit0024] Hagger, M. S. , & Chatzisarantis, N. L. D. (2007). *Intrinsic motivation and self-determination in exercise and sport. Champaign* . Human Kinetics.

[cit0025] Hausenblas, H. A. , & Fallon, E. A. (2006). Exercise and body image: A meta-analysis. *Psychology and Health* , *21* (1), 33–47. 10.1080/14768320500105270

[cit0026] Hills, A. P. , Dengel, D. R. , & Lubans, D. R. (2015). Supporting public health priorities: Recommendations for physical education and physical activity promotion in schools. *Progress in cardiovascular diseases* , *57* (4), 368–374. 10.1016/j.pcad.2014.09.010 25269062

[cit0027] Imbong, J. A. M. (2019). Book review: Deborah lupton, the quantified self: A sociology of self-tracking. *Mobile Media & Communication* , *7* (1), 151–152. 10.1177/2050157918804400a

[cit0028] Irwin, J. D. (2004). Prevalence of university students’ sufficient physical activity: A systematic review. *Perceptual and Motor Skills* , *98* (3), 927–943. 10.2466/pms.98.3.927-943 15209309

[cit0029] Keating, X. D. , Guan, J. , Piñero, J. C. , & Bridges, D. M. (2005). A meta-analysis of college students’ physical activity behaviors. *Journal of American College Health* , *54* (2), 116–126. 10.3200/JACH.54.2.116-126 16255324

[cit0030] Kerner, C. , & Goodyear, V. A. (2017). The motivational impact of wearable healthy lifestyle technologies: A self-determination perspective on fitbits with adolescents. *American Journal of Health Education* , *48* (5), 287–297. 10.1080/19325037.2017.1343161

[cit0031] Kirk, D. (2010). *Physical education futures* . London: Routledge.

[cit0032] Kriemler, S. , Meyer, U. , Martin, E. , van Sluijs, E. M. F. , & Andersen, L. B. (2011). Effect of school-based interventions on physical activity and fitness in children and adolescents: A review of reviews and systematic update. *British Journal of Sports Medicine* , *45* (11), 923–930. 10.1136/bjsports-2011-090186 PMC3841814 21836176

[cit0033] Kryeziu, A. R. , Iseni, A. , Teodor, D. F. , Croitoru, H. , & Badau, D. (2023). Effect of 12 weeks of the plyometric training program model on speed and explosive strength abilities in adolescents. *Applied Sciences* , *13* (5), 2776. 10.3390/app13052776

[cit0034] Leslie, E. , Owen, N. , Salmon, J. , Bauman, A. , Sallis, J. F. , & Lo, S. K. (1999). Insufficiently active Australian college students: Perceived personal, social, and environmental influences. *Preventive medicine* , *28* (1), 20–27. 10.1006/pmed.1998.0375 9973584

[cit0035] Lewis, Z. H. , Ottenbacher, K. J. , Fisher, S. R. , Jennings, K. , Brown, A. F. , Swartz, M. C. , & Lyons, E. J. (2016). Testing activity Monitors' effect on health: Study protocol for a randomized controlled trial among older primary care patients. *JMIR research protocols* , *5* (2), e59. 10.2196/resprot.5454 PMC4867768 27129602

[cit0036] Lubans, D. , Richards, J. , Hillman, C. , Faulkner, G. , Beauchamp, M. , Nilsson, M. , Kelly, P. , Smith, J. , Raine, L. , & Biddle, S. (2016). Physical activity for cognitive and mental health in youth: A systematic review of mechanisms. *Pediatrics* , *138* (3), e20161642. 10.1542/peds.2016-1642 27542849

[cit0037] Lyons, E. J. , Lewis, Z. H. , Mayrsohn, B. G. , & Rowland, J. L. (2014). Behavior change techniques implemented in electronic lifestyle activity monitors: A systematic content analysis. *Journal of medical Internet research* , *16* (8), e192. 10.2196/jmir.3469 PMC4147713 25131661

[cit0038] McKenzie, T. L. , & Lounsbery, M. A. F. (2013). Physical education teacher effectiveness in a public health context. *Research Quarterly for Exercise and Sport* , *84* (4), 419–430. 10.1080/02701367.2013.844025 24592772

[cit0039] Michie, S. , Yardley, L. , West, R. , Patrick, K. , & Greaves, F. (2017). Developing and evaluating digital interventions to promote behavior change in health and health care: Recommendations resulting from an international workshop. *Journal of medical Internet research* , *19* (6), e232. 10.2196/jmir.7126 PMC5509948 28663162

[cit0040] Nelson, M. C. , Story, M. , Larson, N. I. , Neumark-Sztainer, D. , & Lytle, L. A. (2008). Emerging adulthood and college-aged youth: An overlooked age for weight-related behavior change. *Obesity (Silver Spring, Md.)* , *16* (10), 2205–2211. 10.1038/oby.2008.365 18719665

[cit0041] Ntoumanis, N. , Ng, J. Y. Y. , Prestwich, A. , Quested, E. , Hancox, J. E. , Thøgersen-Ntoumani, C. , Deci, E. L. , Ryan, R. M. , Lonsdale, C. , & Williams, G. C. (2021). A meta-analysis of self-determination theory-informed intervention studies in the health domain: Effects on motivation, health behavior, physical, and psychological health. *Health Psychology Review* , *15* (2), 214–244. 10.1080/17437199.2020.1718529 31983293

[cit0042] Patel, M. S. , Asch, D. A. , & Volpp, K. G. (2015). Wearable devices as facilitators, not drivers, of health behavior change. *JAMA : the Journal of the American Medical Association* , *313* (5), 459–460. 10.1001/jama.2014.14781 25569175

[cit0043] Piwek, L. , David, E. , Andrews, S. , & Joinson, A. (2016). The rise of consumer health wearables: Promises and barriers. *PLoS Medicine* , *13* (2), e1001953. 10.1371/journal.pmed.1001953 PMC4737495 26836780

[cit0044] Prince, S. A. , Adamo, K. B. , Hamel, M. E. , Hardt, J. , Connor Gorber, S. , & Tremblay, M. (2008). A comparison of direct versus self-report measures for assessing physical activity in adults: A systematic review. *The international journal of behavioral nutrition and physical activity* , *5* , 56. 10.1186/1479-5868-5-56 PMC2588639 18990237

[cit0045] Rand, T. , Tilga, H. , Abós, [. , García-González, L. , Diloy Peña, S. , Burgueño-Mengibar, R. , & Koka, A. (2026). Evaluating effectiveness of the FiTeens intervention for health behavior change in students: A study protocol. *Methods and Protocols* , *9* (4), 101. 10.3390/mps9040101 PMC13397910 42496553

[cit0046] Rhodes, R. E. , Janssen, I. , Bredin, S. S. D. , Warburton, D. E. R. , & Bauman, A. (2017). Physical activity: Health impact, prevalence, correlates and interventions. *Psychology & health* , *32* (8), 942–975. 10.1080/08870446.2017.1325486 28554222

[cit0047] Ridgers, N. D. , McNarry, M. A. , & Mackintosh, K. A. (2016). Feasibility and effectiveness of using wearable activity trackers in youth: A systematic review. *JMIR mHealth and uHealth* , *4* (4), e129. 10.2196/mhealth.6540 PMC5143467 27881359

[cit0048] Rutterford, C. , Copas, A. , & Eldridge, S. (2015). Methods for sample size determination in cluster randomized trials. *International Journal of Epidemiology* , *44* (3), 1051–1067. 10.1093/ije/dyv113 PMC4521133 26174515

[cit0049] Ryan, R. M. , & Deci, E. L. (2017). *Self-determination theory: Basic psychological needs in motivation, development, and wellness.* The Guilford Press. 10.1521/978.14625/28806

[cit0050] Sallis, J. F. , McKenzie, T. L. , Beets, M. W. , Beighle, A. , Erwin, H. , & Lee, S. (2012). Physical Education’s Role in Public Health: Steps Forward and Backward Over 20 Years and HOPE for the Future. *Research Quarterly* , *83* (2), 125–135. 10.1080/02701367.2012.10599842. PMC6036633 22808697

[cit0051] Selwyn, N. (2016). *Education and technology* . London: Bloomsbury.

[cit0052] Stefanica, V. , Ceylan, H. İ. , Mihailescu, L. , Badau, D. , Joksimović, M. , & Rosu, D. (2025). Impact of CrossFit intervention on mental health and well-being among first-year law students. *Humanities and Social Sciences Communications* , *12* , 971. 10.1057/s41599-025-05311-y

[cit0053] Teixeira, P. J. , Carraça, E. V. , Markland, D. , Silva, M. N. , & Ryan, R. M. (2012). Exercise, physical activity, and self-determination theory: A systematic review. *The international journal of behavioral nutrition and physical activity* , *9* , 78. 10.1186/1479-5868-9-78 PMC3441783 22726453

[cit0054] WHO . (2020). *Guidelines on physical activity* . Geneva: WHO.

