# Supplementary material for: From Gym to App: integrating digital monitoring to enhance student engagement in physical education
Source: Health Psychol Behav Med. 2026 Oct 14;14(1):2741524. doi: 10.1080/21642850.2026.2741524 (PMC13670276; doi:10.1080/21642850.2026.2741524)
Supplement: Supplementary file 1 [file RHPB_A_2741524_SM1220.pdf]

Transilvania
University
of Brasov

Bd. Eroilor 29
500036, Brașov
Tel.: +40 268 413 000
rectorat@unitbv.ro / www.unitbv.ro

No. 148/25.01.2025

TRANSILVANIA UNIVERSITY OF BRASOV
FACULTY OF PHYSICAL EDUCATION AND MOUNTAIN SPORTS

ETHICS COMMISSION OF RESEARCH

The Research Ethics Commission from the Faculty ot Physical Education and Mountain Sports, met on 25th of January 2025, discussed the request regarding the approval of the research work „From Gym to App: Integrating Digital Monitoring to Enhance Student Engagement in Physical Education".

The blank informed consent for each subject was signed individual, in romanian language. Each of the subjects agreed to participate in the study and to publish the results, with the mention that their names will not be made public.

25.01.2025

President of the ethics committee and Dean of the faculty
Associate Professor PhD
Ioan Turcu

ROMÂNIA
UNIVERSITATEA TRANSILVANIA DIN BRAȘOV
FACULTATEA DE EDUCAȚIE FIZICĂ ȘI SPORTURI MONTANE
MINISTERUL EDUCAȚIEI
